# [Corrigendum] Efficacy of transarterial chemoembolization-hepatic arterial infusion chemotherapy combined with targeted therapy and immunotherapy in hepatocellular carcinoma with portal vein tumor thrombosis 

**DOI:** 10.3892/ol.2026.15473

**Published:** 2026-01-28

**Authors:** Xunbo Hou, Qiannan Xu, Linan Yin, Huiwen Wang, Juan Wu, Bowen Liu, Dongfeng He, Ruibao Liu

Oncol Lett 30: 363, 2025; DOI: 10.3892/ol.2025.15109

Subsequently to the publication of the above paper, the authors have contacted the Editrial Office to explain that, due to an inadvertent file upload error made during the submission process, Table I contained incorrect data regarding treatment-related adverse events in Group 1 (n=16 patients). The corrected version of Table I is shown on the next two pages, and the correct data for the Treatment-related adverse events in Group 1 column are highlighted in bold.

As a result of the inclusion of the incorrect data in Table I, the corresponding paragraph in the Results section (p. 3, the right-hand column) also contained inaccuracies. The text starting from line 36 should have as follows: “Treatment-related adverse events (AEs) were significantly more frequent and severe in Group 1 compared with the other groups **(all P<0.001)**. In Group 1 (n=16), the most common adverse events included nausea and vomiting (**14** **patients, 87.5%**), weight loss (**13** **patients, 81.2%**), abdominal pain (**12** **patients, 75.0%**), decreased appetite (**12** **patients, 75.0%**), diarrhea (**11** **patients, 68.8%**), rash (**11** **patients, 68.8%**), fatigue (**9** **patients, 56.2%)**, hand-foot syndrome (**6** **patients, 37.5%**), immune-related pneumonitis (**5** **patients, 31.2%**), and hemorrhagic (bleeding) events (**5** **patients, 31.2%**). Oral mucositis occurred in **7** **patients (43.8%).** No treatment-related deaths were observed in any group.”

Finally, the authors requested that an update be made to the corresponding author's email address for long-term academic correspondence. Although the originally listed email (ruibaoliu111@sina.com) remains active, Dr Ruibao Liu now uses **liu_ruibao@sina.com** as his primary and stable professional contact address, and the authors request that this email address should be used for all professional correspondence in the future.

Note that all the other data in the manuscript, including the baseline characteristics (for example, ascites, hepatitis C status and tumor burden), laboratory values, survival outcomes and statistical conclusions remain accurate and unchanged. The authors wished to confirm that the main findings and clinical implications of the study are unaffected by the errors that were made in Table I. The authors regret that these errors went unnoticed prior to the publication of this paper, and apologize to the readership for any inconvenience caused.

## Figures and Tables

**Table I.** Clinical information of patients with hepatocellular carcinoma in the four treatment groups.

Characteristic	Group 1	Group 2	Group 3	Group 4	P-value	Between-group variations
Age, years	53.38±7.24	54.07±10.02	57.49±8.59	53.58±9.35	0.024	NS
Sex, n (%)					0.018	NS
Male	15 (93.75)	77 (85.6)	87 (85.3)	28 (65.12)		
Female	1 (6.25)	13 (14.4)	15 (14.7)	15 (34.88)		
Preoperative indices						
AST, U/l	54.00	52.00	53.00	54.00	0.926	NS
	(42.25, 102.50)	(35.50, 91.50)	(38.00, 82.25)	(39.00, 83.00)		
ALT, U/l	40.50	35.00	36.50	37.00	0.955	NS
	(25.00, 53.25)	(26.00, 63.00)	(24.00, 57.75)	(26.00, 52.00)		
TB, µmol/l	20.30	20.40	20.35	20.10	0.961	NS
	(13.40, 26.25)	(13.90, 26.60)	(13.68, 28.38)	(16.90, 28.10)		
PT, sec	12.20	12.00	12.30	12.50	0.167	NS
	(11.65, 12.88)	(11.35, 12.95)	(11.78, 13.00)	(11.90, 13.10)		
Albumin, g/l	40.90	39.45	37.65	39.50	0.01	Group 2 vs. group 3, P=0.009
	(36.93, 42.10)	(35.80, 43.30)	(34.28, 40.80)	(36.00, 42.30)		
Tumor count, n (%)					<0.001	Group 1 vs. group 2, P=0.0047
≤3	6 (37.5)	5 (5.6)	96 (94.1)	27 (62.8)		
>3	10 (62.5)	85 (94.4)	6 (5.9)	16 (37.2)		
Maximum tumor diameter, n (%)					<0.001	Group 1 vs. group 2, P<0.001
<100 mm	8 (50.0)	0 (0.0)	62 (60.8)	33 (76.7)		
≥100 mm	8 (50.0)	90 (100.0)	40 (39.2)	10 (23.3)		
Medical history, n (%)						
Hepatitis B	4 (25.0)	70 (77.8)	73 (71.6)	34 (79.1)	<0.001	Group 1 vs. group 2, P<0.001
Hepatitis C	15 (93.8)	4 (4.4)	15 (14.7)	3 (7.00)	<0.001	Group 1 vs. group 2. P<0.001
Cirrhosis	4 (25.0)	67 (74.4)	75 (73.5)	33 (76.7)	<0.001	Group 1 vs. group 2, P<0.001
Ascites	13 (81.3)	18 (20.0)	34 (33.3)	2 (4.7)	<0.001	Group 1 vs. group 2, P<0.001
ECOG, n (%)					0.285	NS
<1	12 (75.0)	51 (56.7)	51 (50.0)	32 (74.4)		
≥1	4 (25.0)	39 (43.3)	51 (50.0)	11 (25.6)		
Child-Pugh classification					<0.001	Group 2 vs. group 3, P<0.001
A	14 (87.5)	90 (100.0)	80 (78.4)	39 (90.7)		
B	2 (12.5)	0 (0.0)	22 (21.6)	4 (9.3)		
Pre-treatment AFP, n (%)					0.299	NS
<400 ng/ml	7 (43.8)	46 (51.1)	54 (52.9)	16 (38.1)		
≥400 ng/ml	9 (56.2)	44 (48.9)	48 (47.1)	27 (61.9)		
**Treatment-associated adverse events, n=16 (%)**						
Fatigue	**9 (56.2)**	6 (6.7)	46 (45.1)	5 (11.6)	<0.001	Group 1 vs. group 2, P<0.001
Abdominal pain	**12 (75.0)**	0 (0.0)	42 (41.2)	2 (4.7)	<0.001	Group 1 vs. group 2, P<0.001
Diarrhea	**11 (68.8)**	7 (7.8)	39 (38.2)	2 (4.7)	<0.001	Group 1 vs. group 2, P<0.001
Nausea and vomiting	**14 (87.5)**	5 (5.6)	37 (36.3)	5 (11.6)	<0.001	Group 1 vs. group 2, P<0.001
Decreased appetite	**12 (75.0)**	7 (7.8)	42 (41.2)	8 (18.6)	<0.001	Group 1 vs. group 2, P<0.001
Weight loss	**13 (81.2)**	7 (7.8)	49 (48.0)	8 (18.6)	<0.001	Group 1 vs. group 2, P<0.001
Rash	11 (68.8)	8 (8.9)	26 (25.5)	0 (0.0)	<0.001	Group 1 vs. group 2, P<0.001
Oral mucositis	**7 (43.8)**	6 (6.7)	12 (11.8)	0 (0.0)	<0.001	Group 1 vs. group 2, P<0.001
Hand-foot syndrome	**6 (37.5)**	2 (2.2)	14 (13.7)	0 (0.0)	<0.001	Group 1 vs. group 2, P<0.001
Joint pain	**8 (5.0)**	2 (2.2)	9 (8.8)	0 (0.0)	<0.001	Group 1 vs. group 2, P<0.001
Bleeding events	**5 (31.2)**	4 (4.4)	30 (29.4)	0 (0.0)	<0.001	Group 1 vs. group 2, P<0.001
Immune-related pneumonitis	**5 (31.2)**	2 (2.2)	9 (8.8)	0 (0.0)	<0.001	Group 1 vs. group 2, P<0.001

AST, aspartate aminotransferase; ALT, alanine aminotransferase; TB, total bilirubin; PT, prothrombin time; ECOG, Eastern Cooperative Oncology Group; AFP, α-fetoprotein; NS, no significance.

